# Sequencing and analysis of the complete mitochondrial genome of *Culex gelidus* (Diptera: Culicidae)

**DOI:** 10.1080/23802359.2017.1361350

**Published:** 2017-07-31

**Authors:** Ling Sun, Wen-Bo Fu, Zhen-Tian Yan, Ting-Jing Li, Yi-Ran Ding, Bin Chen

**Affiliations:** Chongqing Key Laboratory of Vector Insects; Institute of Entomology and Molecular Biology, Chongqing Normal University, Chongqing, P.R. China

**Keywords:** Mitochondrial genome, *Culex gelidus*, sequence analysis, Culicidae, phylogeny

## Abstract

In this study, we sequenced and analyzed the complete mitochondrial genome of *Culex gelidus.* The mitogenome is 15,600 bp long, and contains 13 protein-coding genes (PCGs), 22 tRNA genes, two rRNA genes, and a control region. The gene order and composition are identical with those mitogenomes reported in other mosquito species. The whole base composition is A (39.8%), T (39.2%), G (8.8%), and C (12.2%). The PCGs have the initiation codon ATN except for *COI* with a TCG, and possess the complete termination codon TAA or incomplete T. The phylogenetic analysis of known *Culex* mitogenome sequences was carried out based on the nucleotide sequences of 13 PCGs, and the result showed that *Cx. P. pipiens*, *Cx. P. pallens,* and *Cx. p. quinquefasciatus* were claded into the Pipiens Complex in the Pipiens Group, and *Cx. gelidus* might be inappropriate to be classified into the Pipiens Group. Whether the mosquito species group with *Cx. gelidus* as type should be regarded as a Gelidus Group or Subgroup and its taxonomic position need to be elucidated with more molecular data.

*Culex gelidus* is an important vector of Japanese encephalitis virus and can potentially transmit West Nile, Kunjin, Murray Valley encephalitis, and Ross River viruses (Sudeep et al. 2015). It is mainly distributed in India and Southeast Asia (Sudeep et al. 2015), and its taxonomic position has long been disputed (Lu 1997; Harbach 2011; Harbach et al. 2012). The genus *Culex* is cosmopolitan distribution and its many species are important vectors of mosquito-borne diseases, including epidemic encephalitis, and lymphatic filariasis (Lu 1997). The phylogeny of the *Culex* is still unsettled (Harbach 2011), and the knowledge of mitochondrial genome (mitogenome) in the genus is quite limited. Up to date, there have been four species or subspecies with mitogenome sequences available in GenBank in the genus, *Cx*. *P*. *pipiens*, *Cx*. *Quinquefasciatus*, *Cx*. *tritaeniorhynchus,* and *Cx*. *p*. *pallens*. There are four mitogenome sequences for *Cx. p. pipiens* from different populations (three of these completely identical), one for *Cx. p. pallens*, and two for each of *Cx. quinquefasciatus* and *Cx. tritaeniorhynchu* with mutual nucleotide sequence differences. In addition, the mitogenome sequences of four other species (*Cx. camposi*, *Cx. coronator*, *Cx. usquatus,* and *Cx. usquatissimus*) in the genus have also been reported but their sequences are still unavailable in GenBank (Demari-Silva et al. 2015).

For mitogenome, the nucleotide sequences of protein-coding genes (PCGs) are thought to be most suitable as markers to elucidate the phylogenetic relationships in the genus (Luo et al. 2016). In the paper, we report the complete mitochondrial genome of *Cx. gelidus*, and reconstructed the phylogeny of species/subspecies using the PCG nucleotide sequences of seven known mitogenome sequences.

Using the method from Zou et al. (2015), the total DNA was extracted from the species of mosquitoes, which were collected from Shangsi County, Guangxi Province, China (22° 9.28′N, 108° 08.58′E). The total DNA was stored at −80 °C refrigerator in Institute of Entomology and Molecular Biology, Chongqing Normal University. The All polymerase chain reactions were carried out as described in Luo et al. (2016). Eighteen primer pairs (Zhang et al. 2013) were used for the amplification of the complete mitogenome.

The complete mitogenome of *Cx. gelidus* is a double-stranded circular molecule of 15 600 bp in size, containing 13 PCGs, 22 tRNA genes, two rRNA genes, and a control region (CR). The whole nucleotide composition is 39.8% A, 39.2% T, 8.8% G, and 12.2% C, presenting an obvious A + T bias (79.0%). All genes of the mitogenome are encoded on heavy strand except for four PCGs genes (*ND1*, *ND4*, *ND4L*, and *ND5*), nine tRNA genes (*trnQ*, *trnC*, *trnY*, *trnS*, *trnF*, *trnH*, *trnP*, *trnL*, and *trnV*) and two rRNA genes. Except for *COI* starting with TCG, other PCGs use ATG, ATA or ATC as the initiation codon. Eight PCGs (*ND2*, *APT8*, *ATP6*, *COIII*, *ND3*, *ND5*, *ND6*, and *Cytb*) stop with the complete terminate codon TAA, and the rest have incomplete stop codon T. All tRNAs vary from 66 to 72 bp in length. The overall AT content of 22 tRNAs is 79.6%. Among the 22 tRNAs, *trnE* has the highest AT content 90.9% and *trnR* has the lowest AT content 67.2%. There are two rRNAs in the entire mitogenome of *Cx. gelidus*, 16S rRNA with a length of 1338 bp and 12S rRNA with a length of 805 bp. The 16S rRNA is assumed to fill up the blanks between *trnL* and *trnV*, and the 12S rRNA is located between *trnV* and the CR. The CR is 722 bp long with a high AT content 90.6%. We identified two tandem repeat sequences in the CR, the 18 bp of conservative T-stretch region, and the 87 bp of sequence duplicated from the AT unit, (AT)_n_.

We constructed the Maximum Likelihood (ML) tree of *Cx. gelidus* and seven other *Culex* mitogenome sequences available in NCBI based on the nucleotide sequences of the 13 PCGs, with the *An. Gambiae* as outgroup. As shown in the phylogenetic tree (Figure 1), the eight *Culex* mitogenome sequences were divided into three clades: the Pipiens Complex clade with five species/subspecies and 100% of bootstrap support, the Sitiens Group clade with only two mitogenomes of *Cx. tritaeniorhynchus* and supported by a 100% bootstrap, and the *Cx. gelidus* clade with an only 50% bootstrap support to group with Sitiens Group. In the Pipiens Complex clade, there is a quite small genetic distance (0.00010) between *Cx. p. pallens* and *Cx. pipiens* from Turkey with 92% of bootstrap support. The separation of two mitogenomes of *Cx. p. quinquefasciatus* were located between *Cx. p. pallens* and *Cx. p. pipiens* with a non-significant supported bootstrap of 49% and a genetic distance of 0.00063. The *Cx. p. pipiens* was linked to other mitogenome sequences in the Pipiens Complex clade with 100% of bootstrap support and a genetic distance of 0.01182. The relationships of the five mitogenome sequences in the Pipiens Complex clade are consistent with the previous study (Luo et al. 2016), and it further demonstrates that *Cx. p. pallens*, *Cx. p. pipiens,* and *Cx. p. quinquefasciatus* were in a monophyly and they should have the same taxonomic status.

**Figure 1.**
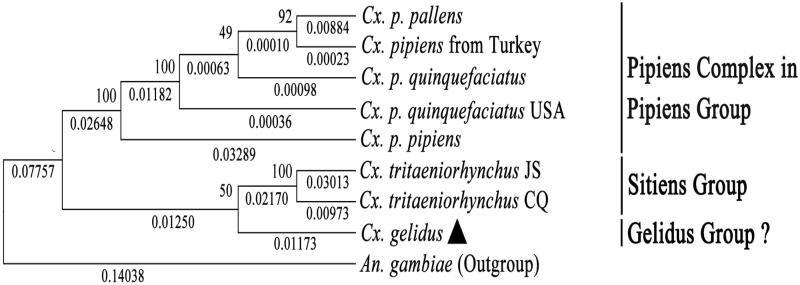
The ML tree was constructed using the nucleotide sequences of the 13 PCGs of the *Cx. gelidus* and seven other *Culex* mitogenome sequences available in GenBank. The best DNA evolution model, GTR + G + I, detected by Model Test was used for the construction of the phylogenetic tree. The bootstrap values for 1000 replicates were indicated above each node of the tree, and the phylogenetic distances were marked beneath each node. Species/subspecies with GenBank mitogenome accession numbers in bracket: *Cx. p. pallens* (KT851543.1), *Cx. pipiens* from Turkey (HQ724616.1), *Cx. p. quinquefasciatus* (GU188856.1), *Cx. p. quinquefasciatus* from USA (HQ724617.1), *Cx. p. pipiens* (NC_-_015079.1), *Cx. tritaeniorhynchus* JS (NC_-_028616.1), *Cx. tritaeniorhynchus* CQ (KT852976.1), *Cx. gelidus* (KX753344), *An. gambiae* (NC_-_002084).

The *Cx. gelidus* as a type species was a classified to an independent group, Gelidus Group, based on the morphological characteristics (Lu 1997), and subsequently Gelidus Group was degraded as the Gelidus Subgroup, designated to Pipiens Group (Harbach 2011), and Sitiens Group (Harbach et al. 2012), respectively, both based on morphological characteristics. In the phylogenetic analysis based *COI* sequences, *Cx. gelidus* and *Cx. sitiens* (Sitiens Group) were claded with a low bootstrap value, 24% (Wang et al. 2012). In the present study, *Cx. gelidus* seemed to have closer relationship with *Cx. tritaeniorhynchus* (genetic distance 0.02170) than Pipiens Complex (genetic distance 0.02648 + 0.01250); therefore, *Cx. gelidus* might be inappropriate to be classified into the Pipiens Group. *Cx. gelidus* was grouped into the *Cx. tritaeniorhynchus* clade, but with only 50% bootstrap support and 0.01250 of genetic distance. Due to only two species (*Cx. tritaeniorhynchus* and *Cx. gelidus*) included in the analysis in the Sitiens Group + *Cx. gelidus* clade, whether the mosquito species with *Cx. gelidus* as type should be regarded as a Gelidus Group or Subgroup and its taxonomic position need to be elucidated with more molecular data.
